# New Approaches to Multi-Parametric HIV-1 Genetics Using Multiple Displacement Amplification: Determining the What, How, and Where of the HIV-1 Reservoir

**DOI:** 10.3390/v13122475

**Published:** 2021-12-10

**Authors:** Sean C. Patro, Aurelie Niyongabo, Frank Maldarelli, Mary F. Kearney

**Affiliations:** HIV Dynamics and Replication Program, National Cancer Institute, Frederick, MD 21702, USA; aurelieniyongabo@gmail.com (A.N.); fmalli@mail.nih.gov (F.M.); kearneym@mail.nih.gov (M.F.K.)

**Keywords:** HIV reservoir, multiple displacement amplification, integration sites analysis, near full-length genome amplification, intact proviral genomes, clonal expansion

## Abstract

Development of potential HIV-1 curative interventions requires accurate characterization of the proviral reservoir, defined as host-integrated viral DNA genomes that drive rebound of viremia upon halting ART (antiretroviral therapy). Evaluation of such interventions necessitates methods capable of pinpointing the rare, genetically intact, replication-competent proviruses within a background of defective proviruses. This evaluation can be achieved by identifying the distinct integration sites of intact proviruses within host genomes and monitoring the dynamics of these proviruses and host cell lineages over longitudinal sampling. Until recently, molecular genetic approaches at the single proviral level have been generally limited to one of a few metrics, such as proviral genome sequence/intactness, host-proviral integration site, or replication competency. New approaches, taking advantage of MDA (multiple displacement amplification) for WGA (whole genome amplification), have enabled multiparametric proviral characterization at the single-genome level, including proviral genome sequence, host-proviral integration site, and phenotypic characterization of the host cell lineage, such as CD4 memory subset and antigen specificity. In this review, we will examine the workflow of MDA-augmented molecular genetic approaches to study the HIV-1 reservoir, highlighting technical advantages and flexibility. We focus on a collection of recent studies in which investigators have used these approaches to comprehensively characterize intact and defective proviruses from donors on ART, investigate mechanisms of elite control, and define cell lineage identity and antigen specificity of infected CD4+ T cell clones. The highlighted studies exemplify how these approaches and their future iterations will be key in defining the targets and evaluating the impacts of HIV curative interventions.

## 1. Background

Treatment of HIV with combination antiretroviral therapy (ART) suppresses plasma viremia below the limit of detection of commercial viral load assays. However, due to the maintenance of a stable HIV reservoir, upon cessation of ART, viremia rebounds to pre-therapy levels in most individuals [1,2,3,4]. The HIV reservoir is defined as the viral genomes integrated into the DNA of host cells (proviruses) [5,6,7] that propagate the recrudescence of viremia upon cessation of ART [5,8,9,10,11,12,13,14]. Achieving HIV remission without ART necessitates the detailed characterization of HIV proviruses that persist on ART and the cells that harbor them to understand their maintenance and to reveal potential targets for future efforts to eradicate or suppress rebound viremia [15,16,17].

Due to frequent errors in HIV replication [7,18,19,20,21] and to strong selection by host immune responses [22,23,24,25], genetically intact proviruses comprise only 0.5–5% of all proviral sequences that persist during ART [26,27,28]. Estimates of the HIV reservoir size [29] have been guided by quantitative viral outgrowth assays (VOA) [26,30,31,32,33,34] that detect and quantify intact proviruses in cell samples by inducing them to express virus particles in limiting-dilution culture wells ex vivo or mice in vivo [35]. While laborious, these approaches are the gold standard of HIV reservoir quantification. Newer and less laborious PCR and sequencing-based approaches to quantify the reservoir attempt to amplify the near full-length (NFL) HIV genome or regions of the genome that are commonly deleted, including the near full-length single-genome sequencing assay (NFL-SGS) [26,27,36,37,38], the intact proviral DNA assay (IPDA) [28], the quadruplex qPCR (Q4PCR) assay [39], the tat/rev induced limiting dilution assay (TILDA) [40], and others [34,41]. A proverbial needle in a haystack, detecting genetically intact proviruses necessitates large amounts of donor material (e.g., blood, lymph node). Complicating the characterization of the HIV reservoir is the fact that, once observed (e.g., in a viral outgrowth or PCR-based assay), the original single HIV genome is typically unrecoverable and, therefore, removed from further study.

In addition to quantifying the HIV reservoir, HIV genomes that persist during ART have been genetically characterized using a variety of molecular approaches. For example, combining fluorescence-activated cell sorting (FACS) with HIV single-genome sequencing (SGS) or integration site analysis (ISA) informs proviral structures (defined as the HIV sequence including deletions, hypermutation, and inversions), the clonality of infected cells, and the integration sites across cell subsets/tissue [37,42]. Capture and enrichment approaches, including G&T-seq for single-cell paired genome and transcriptome characterization [43], HIV SortSeq for infected, single-cell transcriptional analysis [44], HIV-DNA-capture-seq for bulk sequencing of HIV genomes and recovery of some integration sites [45], and PCIP-seq (Pooled CRISPR Inverse PCR sequencing) for linking HIV integration sites to their proviral structures [46] have all been used for multiparametric characterization of HIV. While powerful techniques, these multimodal workflows are limited throughput, in their resolution of single proviral genomes in heterologous samples, and/or their full HIV-genome sequence coverage.

Here, we discuss how the addition of multiple displacement amplification (MDA) of single HIV proviral genomes in a background of host genomic DNA can augment some of the above molecular approaches and allow for more comprehensive and simultaneous characterization of the HIV reservoir. For example, MDA can be performed on proviral endpoint diluted DNA extracted from cells sorted on immunologic markers or collected using other upstream approaches. The MDA-expanded proviruses can then be used for multiple downstream analyses such as SGS and ISA to determine the HIV proviral structure and the site of integration into the host DNA. These MDA-augmented approaches maintain the original donor genetic material (e.g., DNA from blood, lymph node, gut biopsy), allowing for parallel downstream characterization of single proviruses and infected cell clones. Proviral information derived from MDA approaches also provides bookmarks for tracking dynamics of infected cell clones, which is vital to the development and evaluation of current and future curative strategies.

## 2. Comprehensive Evaluation of HIV-Infected Cell Populations Using MDA Approaches

### 2.1. MDA-Augmented Workflow: Amplification, Screening, and Multi-Parametric Analysis

**Figure 1:**
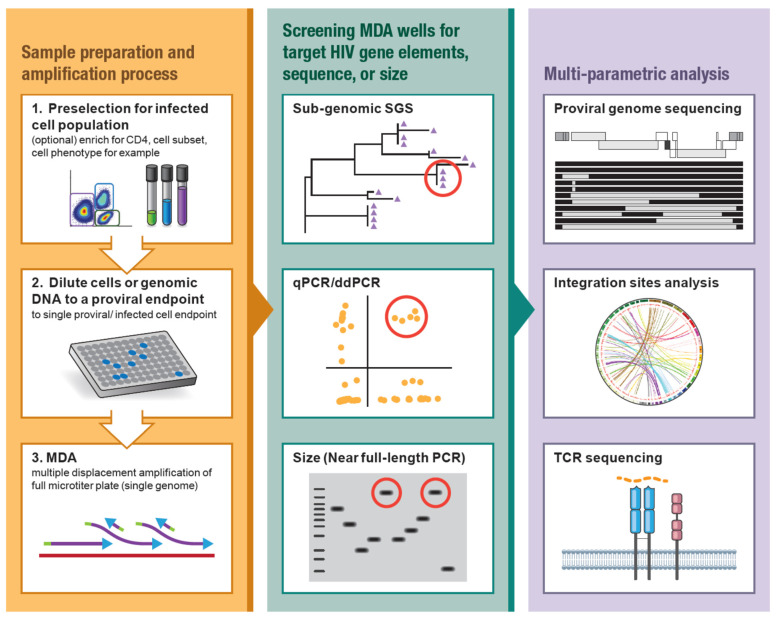
Workflow of MDA-supplemented HIV proviral characterization. (Left) HIV (+) PBMC or other material can be CD4-enriched or cell sorted to target specific CD4+ memory subsets or phenotypes such as activation state or antigen reactivity. Genomic DNA or sorted cells are diluted to a single proviral endpoint and used for multiple displacement amplification (MDA). (Center) MDA reactions are screened for HIV-1 proviruses by amplification and sequencing of a sub-genomic region of HIV (e.g., LTR, *gag*, *int*, *env*), qPCR/ddPCR targeting specific genome elements, or size selection of the near full-length (NFL) HIV amplicon. Red circles designate candidate proviruses for follow-up. (Right) Multi-parametric HIV proviral analysis including full-length (NFL) HIV amplification and sequencing, integration site analysis, host-HIV amplification and sequencing, and/or CD4+ TCR sequencing.

MDA has been developed and optimized for the amplification of a single proviral genome and its flanking host genomic DNA in the background of uninfected cell DNA [47,48]. MDA offers an isothermal, non-templated, non-biased amplification of genetic material within a given sample [49,50,51,52], including the integrated HIV provirus, the host genome, and the proviral-host junctions. Briefly, genomic DNA is extracted from donor material (e.g., PBMC, CD4+ T cells, lymph node biopsies) and diluted to a proviral endpoint such that less than 30% of aliquots contain a single HIV provirus. The MDA reaction, commercially available or lab-optimized [49,50,51,52], utilizes phi29 DNA polymerase with random oligomers for unbiased, isothermal (30 °C) amplification. The branching DNA polymerization reaction generates greater than 10 kb fragments that include a 100 to 10,000-fold amplification of the HIV provirus in the context of its host genome [10,11]. The resulting MDA reactions contain many copies of the HIV proviruses that can then be used for multiple downstream applications (Figure 1). Figure 1 highlights a generic workflow of MDA-augmented HIV proviral characterization. Upstream of MDA, donor cells can be enriched for CD4+ T cells or FACS-sorted to investigate distinct CD4+ memory subsets, activation phenotypes, or CD4+ T cells reactive to specific antigens. Downstream from MDA, aliquots can be screened for HIV proviruses by PCR amplification and sequencing of sub-genomic regions of HIV (e.g., LTR, *gag*, *pol,* or *env*). The sequence information resulting from the screens can then be used to identify proviruses of interest by pairing to previous sequence datasets [11,53]. In addition, MDA products can be screened for HIV using quantitative PCR or droplet digital PCR that target specific genome elements, such as those described in the IPDA and Q4PCR assays [28,39]. MDA wells that screen positive for HIV can be used for NFL-SGS, allowing for identification of proviruses that are inferred intact [26,27,37,38]. Downstream analysis of MDA aliquots may also include integration site analyses [5,6,8,54,55] and CD4+ TCRβ sequencing, further informing T cell clonality [14].

### 2.2. Persistence of Clonally Expanded, Infected CD4+ T-Cells with Intact and Defective Proviruses in Individuals on Suppressive ART

Clonal expansion of infected CD4+ T-cells was first suggested by observation of identical sub-genomic and NFL sequences in plasma and PBMC but was confirmed by the detection of identical proviral integration sites in ART-treated individuals [5,6,8,27,37,38,42,53,56,57,58,59,60,61]. Identical viral sequences observed in rebounding viruses during analytical treatment interruptions (ATI) and in outgrowth from multiple wells of VOA supported that expansion of infected cells during ART included CD4+ T cells carrying genetically intact, replication-competent proviruses [3,9,32,62,63,64]. The first confirmed replication-competent provirus within an expanded cell clone (AMBI-1) was comprehensively characterized using a combination of integration site analysis, genomic DNA and plasma NFL-SGS, amplification and sequencing with host-proviral junction primers, and VOA [5,8]. Despite a change in ART regimen, the wild-type AMBI-1 viral genome persisted in plasma and PBMC for years on ART [8].

Observing clusters of proviral populations with sub-genomic identity in donors on ART, Patro et al. used MDA followed by single-genome sequencing and integration sites analysis to address if such groups resulted from infected cell clones (identical integration sites) or from multiple cells being infected with identical or similar proviruses (different integration sites) (n = 5 donors, n = 10 clusters of identical sequences) [11]. Proviruses in these clusters with identical integration sites (cell clones) were identified in all donors. Although identical sub-genomic sequences often corresponded to identical integration sites, many clusters also included singly observed integration sites distinct from the integration site in the cell clone. The authors suggested that genetic bottlenecks occurring before ART may be another source of proviruses with sub-genomic identity, evidenced in proviral populations with drug-resistance mutations and in those with low genetic diversity/early treatment (i.e., transmission bottleneck) [61,65]. In one proviral population with low genetic diversity, although no intact sequences were observed, reconstruction of the intact viral ancestor could be inferred from proviruses with non-overlapping deletions. Albeit indirectly, such an approach could be useful for estimating the sequences of the intact proviruses that persist on ART in those with small proviral reservoirs or for inferring the transmitted/founder lineages that initiate HIV infections [65].

Einkauf et al. developed an MDA approach called MIP-Seq (matched integration site and proviral sequencing) to interrogate the contribution of integration sites to the persistence of intact proviruses on ART (n = 3 donors) [10]. Their MIP-Seq analyses resulted in 100 intact proviruses (73 unique proviral/IS pairs) and 84 defective proviruses (76 unique proviral/IS pairs) that were used to compare their sites of integration into the human genome. The integration sites of intact proviruses, relative to defective, trended towards enrichment in intergenic/pseudogenic regions (*p* = 0.07), and, in those with genic integration sites, a higher number of gene-opposite integrations were observed for intact relative to defective proviruses (*p* = 0.05). The authors also paired their MIP-Seq analyses with autologous RNA-seq and ATAC-seq approaches, mapping the proximity of transcriptional start sites (TSS), accessible chromatic regions, and nearby transcriptional activity to the integration sites of intact relative to defective proviruses. They found that intact proviruses (relative to defective) in one donor trended toward more distance to transcriptional start sites, open chromatin, and gene expression, supporting a transcriptional silencing model of HIV persistence. For donors 2 and 3, however, intact proviruses were found to be closer to transcriptional start sites and ATAC-seq peaks and trended toward regions of higher local gene expression, supporting a role of transcriptional interference in the maintenance and persistence of these proviral populations. The authors’ contrasting findings across three donors highlight the need for deep characterization of intact proviruses across larger cohorts. Depositing into well-maintained databases, such as the Retroviral Integration Database (https://rid.ncifcrf.gov/, accessed on 8 December 2021) [54] and Proviral Sequence Database (https://psd.cancer.gov/, accessed on 8 December 2021) [55], will allow for statistically powered, cross-study analyses to investigate mechanisms that drive persistence in the majority of proviruses and donors. Complementing the Einkauf 2019 study, Garcia-Broncano et al. also utilized MIP-Seq to characterize intact proviruses and their associated integration sites in neonates born with HIV and initiated on ART days after birth [66].

Both Einkauf et al. and Patro et al. observed cell clones containing intact proviruses, including one that was highly expanded (n = 20 observations) [10] and several that matched or closely matched viruses from VOA experiments, supporting the role of clonal expansion in the persistence of the HIV reservoir. The investigations described above serve as proof-of-concept that MDA-based approaches can be adapted to HIV genetics [10,11], supplementing more laborious methods and allowing for the comprehensive characterization of persistent, intact proviruses within expanded cell clones.

### 2.3. Persistent Plasma Viremia Driven by Clonal Expansion of Cells Containing Intact Proviruses (“Repliclones”) in Individuals on Continuous ART

The case study describing the AMBI-1 cell clone provided a paradigm for the comprehensive characterization of replication-competent proviruses within expanded cell clones as the source of persistent, low-level viremia in donors on ART [5,8]. Building on this model, Halvas et al. investigated a longitudinal cohort with low-level clinically detectable persistent viremia, that is, HIV (+) donors adhering to ART with no evidence of drug-resistance, and with greater than 6 months of non-suppressible plasma viremia (>20 copies/mL) [12]. Near full-length (NFL) proviral genomes were recovered from four of the donors that matched plasma and/or VOA virus, suggesting the presence of infected cell clones in peripheral blood capable of producing replication-competent virus particles in vivo. Incorporating an MDA approach paired with integration site analysis (ISA), the proviral sequences and distinct host genome locations were mapped to introns of genes of ABCA11P (chr4), ZNF268 (chr12), MATR3 (chr5), and ZNF721/ABCA11P (chr4). These proviral clones were termed “repliclones” by the authors. Specific integration site-provirus amplifications [10,11] provided integration site confirmation and additional proviral sequence information. Extensive integration site analyses determined that these repliclones comprised 0.03–1.1% of the integrated proviruses examined, consistent with prevailing data that the reservoir is a minute fraction of all integrated proviruses [26,27,28]. The Halvas et al. study demonstrated the origin of a stable reservoir in a directly clinically relevant cohort and provided a model by which infected expanded clones contribute to persistent low-level viremia. Their findings also support previous studies showing that ART intensification may do little to address such viremia in already adherent cohorts [67,68,69]. The demonstration of a clonally expanded “active reservoir,” presumptively capable of quickly rekindling viremia upon therapy cessation or ATI, provides both a challenge and demonstratable target of future therapies. Furthermore, identifying such repliclones will provide future efforts with discrete, trackable targets for the evaluation of potential curative interventions [16,29].

### 2.4. Characteristics of Intact and Defective Proviruses and “Deep Latency” in Elite Control Individuals

Mechanisms of “elite control” (natural control of viremia below commercial limits of detection without ART) have been studied for decades and include select HLA phenotypes [70,71,72], highly functional/polyfunctional cytotoxic T-lymphocytes (CTL) [73,74], innate NK-KIR (natural killer-killer-cell immunoglobulin-like receptor) alleles [75,76], and other correlates [77]. The composition and dynamics of the proviral reservoir in elite controllers have been investigated using full-length sequencing and characterization of replication competent viruses [78], by demonstrating ongoing viral replication and evolution [79,80], and by characterizing infected cell clones [81]. Seeking to differentiate the HIV reservoir in elite controllers (n = 64 donors) vs. in individuals on long-term ART (n = 41 donors), Jiang et al. found an overall lower proviral/intact proviral burden in elite controllers. Furthermore, the intact proviruses in elite controllers had lower genetic diversity and fewer CTL escape mutations than in donors on long-term ART. The authors suggested that the intact proviruses are maintained, at least in part, by clonal proliferation [13], consistent with previous findings [81,82]. Their comprehensive characterization of proviral populations in elite controllers (n = 11) also revealed oligoclonal phylogenetic landscapes, likened to chronic HTLV-1 infection [83] and to the mono/oligoclonal landscapes in vertically infected children on sustained ART [38].

Using MIP-Seq, the integration sites of intact genomes in elite controllers were investigated by Jiang et al. and were found to be in pseudogenic/non-genic regions (45% versus 17.8%) and were flanked by centromeric or satellite host DNA. The authors also noted a significant enrichment of intact proviruses in zinc finger (ZNF) genes (chr4, 18, 19), including the ZNF721/ABCA11P gene also reported by Halvas et al. [12]. RNA-seq, ATAC-seq, and Hi-C-seq were performed in the aforementioned studies on separate aliquots of autologous CD4+ T cells, CD4+ effector memory T cells, and CD4+ central memory T cells and revealed that sites of intact proviruses in elite controllers, relative to those in ART-treated donors, were further from transcriptional start sites and were further from open chromatic regions. Of note, as transcriptional analysis was performed on separate aliquots of cells, it informs global chromatin structures but may not reflect the specific chromatin structure linked to the intact proviruses. These findings suggest the “deep latency” model of HIV persistence in elite controllers resulting from years or decades of immune-mediated elimination of transcriptionally active proviruses. Jiang et al. analyzed over 1 billion PBMC and gut-associated lymphocytes each from two elite controllers and found 0–1 intact proviruses. While viral replication continues in elite controllers [79,80], efficient, extensive, and rapid CTL killing [84,85] may likely tip the balance toward immune control, exemplified as these two cases demonstrate extreme suppression/elimination of the HIV reservoir. These cases provide a potential model and goal for the design of strategies for HIV remission without continued ART.

A developing theme, intact proviruses integrated within ZNF genes have been observed in several studies (Einkauf et al. [10], Halvas et al. [12], Jiang et al. [13]). To further investigate the relationship between integration site and persistence of intact proviruses, Huang et al. used a modified MIP-Seq approach supplemented with Q4PCR [39] on CD4+ T cells from six donors on long-term ART [86]. They observed an enrichment of intact proviruses integrated into ZNF genes, especially those in expanded CD4+ T cell clones with integration sites within Krüppel-associated box (KRAB) domain–containing ZNF genes. They suggest that integration within ZNF genes may be especially permissive for maintaining HIV latency, perhaps even in T cells that are in an activated state.

### 2.5. Knowing the Host Cell: Identifying Antigen Specificity and CD4+ T Cell Memory Subset of Expanded HIV-Infected T-Cell Clones

Accumulating evidence supports the proliferation of infected CD4+ T cells as a key driver of HIV persistence during ART. Proposed underlying mechanisms include homeostatic proliferation of memory cells [8,9,32,37,53,59,87,88], integration into cancer-associated genes promoting survival or proliferation [5,6], and antigenic-driven activation and proliferation of infected cells [14]. The first detected replication-competent provirus within an expanded cell clone (AMBI-1) was hypothesized to be within a tumor-responding T cell clone, as the provirus was found to be prevalent in the squamous cell carcinoma primary tumor and enriched in the metastases [8]. Simonetti et al. used an MDA approach to further address the role of antigen/TCR-driven expansion of infected CD4+ T cells as a mechanism of proviral persistence [14]. Briefly, CD4+ T cells (n = 10 donors) were sorted for their responsiveness to CMV or HIV antigens. The cells were then dispersed into multiple small aliquots and lysed. The genomes from within the lysed cells were MDA-amplified and used for downstream integration site analyses to identify infected cell clones. The MDA wells containing infected cell clones were used to sequence the full-length HIV proviruses and the TCR CDR3β (TCRβ) sequence associated with them. This approach detected 22 antigen-driven infected cell clones, one of which contained an intact provirus integrated into the *FBXO22* gene. The *FBXO22* integrant was found in longitudinal PBMC samples but did not match HIV recovered from VOA. TCRβ-identity was determined for eight antigen-responding infected cell clones (5 CMV, 3 HIV), from which custom proviral-host and TCRβ primers were used to estimate their frequency in the CD4+ T cell population. Interestingly, within three of the CMV-responding clones, only 14–72% of the cells contained HIV proviruses while 92–93% in two other CMV-responding clones were infected. These important findings demonstrate that HIV-infection occurs both before and during or after antigen-driven expansions.

CD4+ T cell subsets are the result of a continuum of phenotype and function in the antigen recognition, expansion, elimination, and memory response effected by the adaptive, cell-mediated immune response. Defined by combinations of surface markers such as CD45RO/RA, CD27, CCR7, and CD57, these subsets can include TN (naïve, naïve-like) TCM/TCTM (central, central transitional memory), TEM/TEMRA (effector memory, effector memory CD45RA-expressing), and T effector [89,90]. Applying integration site and TCRβ determination (as described above) to T cell subsets, Simonetti et al. demonstrated the presence of infected cell clones residing primarily within the TCM and TEM. The composition of infected cells in the subsets was mirrored by the composition of the TCR clonotype, suggesting antigen, immune factors, and/or other exogenous non-viral factors driving the differentiation state of the provirus-containing cell clones [14]. Similarly, Cole et al. developed and applied STIP-seq (Simultaneous TCR, Integration site, and Proviral sequencing), a modification of the HIV Flow assay [91] that combines single-cell p24+ selection with simultaneous TCR, proviral genome, integration site analysis, and memory subset analysis, to comprehensively characterize the proviral landscape in a cohort of ART-treated individuals (n = 8) [92]. Their report described the finding of five genetically intact proviruses, including one integrated in the ZNF gene, ZNF274.

**Figure 2:**
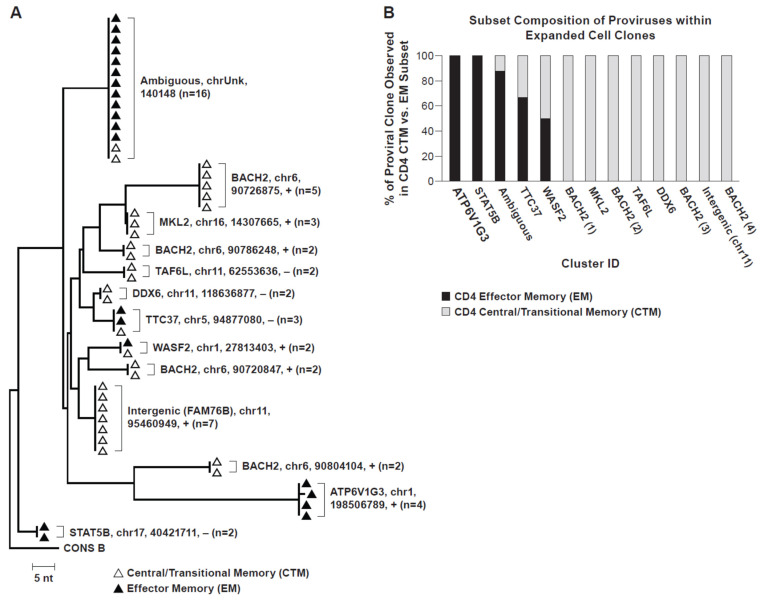
Neighbor-joining trees of MDA-amplified SGS. (**A**) Single-genome sequencing of the P6-PR-RT region in PBMC from Patient 1 [5,8] showing integration-site identical proviruses within expanded CD4+ T cell clones. CD4+ memory subsets from which sequences were recovered are indicated by white (central/transitional memory) or black (effector memory) triangles. Integration site details (gene/nearest gene, chromosome, hg19 location, proviral orientation relative to gene (+/−, with/against, respectively), and number of observations) indicated for each group of identical sequences. (**B**) CD4+ memory T cell subset distribution of identical proviral integration sites within expanded cell clones. Cluster ID designated in coordination with phylogenetic tree (2A).

Using MDA-SGS [11], we also examined the distribution of HIV-infected CD4+ T cell clones within TCM and TEM subsets and found that AMBI-1 and other proviruses in the same individual populated both subsets (Figure 2). These findings are also consistent with observed identical full-length proviral genomes across multiple T cell subsets [37]. Overall, these data suggest that antigen-specific CD4+ T cells that were infected before or during the antigen response are expanded despite containing intact or defective HIV proviruses. The data also suggest that T cells infected with replication-competent proviruses may be capable of returning to a resting state, implying that they are not eliminated via host immunity or viral-mediated cytotoxicity despite activation. The demonstration of antigen-driven expansion of HIV-infected T cell clones and evidence of differentiation across CD4+ memory subsets may present additional challenges to current attempts to achieve HIV remission without ART, such as the “shock and kill” strategies [15,17,93,94]. Future curative strategies may require additional augmentation of host immunity to tip the balance toward host-mediated control and functional cure, as demonstrated naturally in elite controllers.

## 3. Future Directions

Strategies for a functional cure for HIV-1 include “shock and kill” [15,17,93,94], “block and lock” [95,96], enhanced cell-mediated immunity [97,98,99,100,101], engineered “HIV-resistant” T-cells/CRISPR approaches [102,103,104,105,106], and more [107,108]. However, all interventions require an accurate characterization of the target and a means to evaluate the efficacy of the intervention [29]. At current, the gold standards of quantitative VOA and ATI [3,109,110] remain indispensable but require significant time and careful planning. MDA-based approaches have the potential to provide in-depth, multi-parametric characterization of the HIV reservoir during ART and a means to track the dynamics of intact proviruses over time and during curative interventions. To date, MDA approaches have demonstrated: (1) the ability to screen for rare events while maintaining the original source material, (2) the ability to perform parallel analyses of a single provirus or infected cell clone, (3) the ability to identify “repliclones,” including their sites of integration, (4) the ability to track the dynamics of infected cell clones over time, and (5) the ability to characterize infected cells clones, including their memory subset and TCRβ identity. MDA-augmented workflows are not without limitations, however, including: (1) reagents and/or kits are costly compared to other molecular genetic approaches, (2) individual provirus/integration site analyses are labor intensive, and (3) sampling depth (number of proviruses) and breadth (number of donors/timepoints/subsets) is limited due to the cost and labor. Current efforts to automate workflows, target screening to specific proviruses of interest, and reduce costs are underway. As these MDA approaches continue to improve in efficiency and throughput, they will offer significant insights into new potential strategies to achieve HIV remission without ART.

## 4. Methods

### 4.1. Ethics Statement

All participants signed informed consent in accordance with NCT00009256 (AVBIO). Protocols were approved by the internal review boards at the National Institutes of Health.

### 4.2. MDA-SGS

CD4+ memory T cells were sorted into effector and central/transitional memory as in [111] and subjected to MDA-SGS as in [11]. Briefly, genomic DNA was extracted from sorted subsets, diluted to proviral endpoint (such that less than 30% of wells in a microtiter plate are positive for the P6-PR-RT sub-genomic region of HIV-1), and subjected to multiple displacement amplification. Integration sites analysis [5,8,54] was performed on MDA wells containing identical P6-PR-RT sequences.

## Data Deposition

All sequence data reported in this paper are available in GenBank (accession nos. OL343701–OL343752). All integration site data are available in the Retroviral Integration Database [54] (https://rid.ncifcrf.gov/, accessed on 8 December 2021) (accession numbers rid0000001_1–rid0000001_52) or using the PubMed ID of this paper; 0000001 will be replaced with the appropriate PubMed ID once it is granted.

## Manuscript Preparation

Figure 1 was generated with the assistance of BioRender.com. Phylogenetic tree of P6-PR-RT proviral single-genome sequences (Figure 2) was constructed in MEGA7 (https://www.megasoftware.net/, accessed on 8 December 2021) using the neighbor-joining method.
